# Model-Based Analysis of Muscle Strength and EMG-Force Relation with respect to Different Patterns of Motor Unit Loss

**DOI:** 10.1155/2021/5513224

**Published:** 2021-06-22

**Authors:** Chengjun Huang, Maoqi Chen, Yingchun Zhang, Sheng Li, Ping Zhou

**Affiliations:** ^1^Guangdong Work Injury Rehabilitation Center, Guangzhou, China; ^2^Department of Physical Medicine and Rehabilitation, University of Texas Health Science Center at Houston, TX, USA; ^3^TIRR Memorial Hermann Research Center, Houston, TX, USA; ^4^Institute of Rehabilitation Engineering, University of Health and Rehabilitation Sciences, Qingdao, China; ^5^Department of Biomedical Engineering, University of Houston, Houston, TX, USA

## Abstract

This study presents a model-based sensitivity analysis of the strength of voluntary muscle contraction with respect to different patterns of motor unit loss. A motor unit pool model was implemented including simulation of a motor neuron pool, muscle force, and surface electromyogram (EMG) signals. Three different patterns of motor unit loss were simulated, including (1) motor unit loss restricted to the largest ones, (2) motor unit loss restricted to the smallest ones, and (3) motor unit loss without size restriction. The model outputs including muscle force amplitude, variability, and the resultant EMG-force relation were quantified under two different motor neuron firing strategies. It was found that motor unit loss restricted to the largest ones had the most dominant impact on muscle strength and significantly changed the EMG-force relation, while loss restricted to the smallest motor units had a pronounced effect on force variability. These findings provide valuable insight toward our understanding of the neurophysiological mechanisms underlying experimental observations of muscle strength, force control, and EMG-force relation in both normal and pathological conditions.

## 1. Introduction

Voluntary muscle activation is mainly controlled by motor unit recruitment and rate modulation. Neurological disorders can influence motor unit properties, contributing to muscle atrophy, contracture, weakness, unstable force output, and altered electromyogram- (EMG-) force relationship. For example, previous studies have revealed various changes in motor unit properties of poststroke patients, such as loss of functional motor units [1–6], impaired motor unit control properties (reduced motor unit peak firing rates and compressed ranges of motor unit recruitment) [7–10], and altered motor unit morphological features [11–13]. These factors significantly impair muscle force generation and the EMG-force relation. Muscle strength of hemispheric stroke patients is profoundly weaker for the paretic side compared with the contralateral side or neurologically intact subjects. The slope of the EMG-force relation of the paretic first dorsal interosseous and biceps brachii muscles was reported to be significantly greater in hemiparetic stroke survivors compared with the contralateral or neurologically intact muscles [7, 14].

By using an experimental approach, it is difficult to quantify the relative contribution of each of the various motor unit property alterations to muscle weakness and the EMG-force relation. To overcome this difficulty, a classic motor neuron pool model, developed by Fuglevand et al. [15], has been widely used to better understand the experimental observations of the force and EMG signals and explore the mechanisms of motor impairment [16–18]. For example, Shin et al. [19] and Zhou et al. [20] have used the model to explore the effect of motor unit control property (recruitment and firing rate) changes on muscle weakness and the EMG-force relation.

Among different motor unit properties, loss of functional motor units plays a very important role in influencing muscle strength, force variability, and the EMG-force relation. Some studies reported that small motor units (with low recruitment thresholds) were more affected after a neurological injury [13], while others found that large motor units (with high recruitment thresholds) were more susceptible to atrophy [21]. It still remains unclear whether motor unit loss occurred randomly or in a specific pattern. Therefore, the objective of this study was to explore the effects of different patterns of motor unit loss on muscle strength, force variability, and the EMG-force relation. This was performed by varying the model input with three different patterns of motor unit loss, including motor unit loss restricted to the largest ones, motor unit loss restricted to the smallest ones, and motor unit loss without any size restriction.

The Fuglevand model [15] was used in this study. Each pattern of motor unit loss was simulated under two different motor neuron firing strategies. The resultant muscle force amplitude, force variability, and the EMG-force relation under different conditions were compared. The findings of this study can help better understand experimental muscular force and surface EMG recordings in both healthy and pathological conditions and facilitate identification of specific motor unit mechanisms underlying observed alterations in overall muscle force and EMG activities. Such analyses in turn can help design appropriate rehabilitation strategies to improve muscle function by targeting the identified motor unit alterations.

## 2. Methods

There are three main components contained in the motor unit pool model used in this study: a motor neuron pool model, a force generation model, and a surface EMG model [15].

### 2.1. Motor Neuron Pool Model

A motor neuron pool innervating 120 motor units was simulated. Each motor unit had a recruitment threshold (RTE), which is the minimum excitatory drive needed to trigger the motor unit to discharge. The RTE was expressed as an exponential function as Equation (1). In Equation (1), RR is the range of recruitment threshold between the first and last motor units in the pool, *i* is an index identifying each motor unit, and ln is the natural logarithm. According to Equation (1), most of the motor units would be recruited at relatively lower excitation levels. In our study, once the excitatory drive exceeded the RTE, the motor unit started to discharge at a minimum firing rate (MFR) of 8 Hz. RR of the motor unit pool was assigned to be 40% excitation (i.e., the last motor neuron was recruited at 40% maximum excitation).

(1) $$\begin{array}{c} \text{RTE}\left(i\right)=e^{\left(\text{lnRR}/n\right)∙i}. \end{array}$$

**Figure 1:**
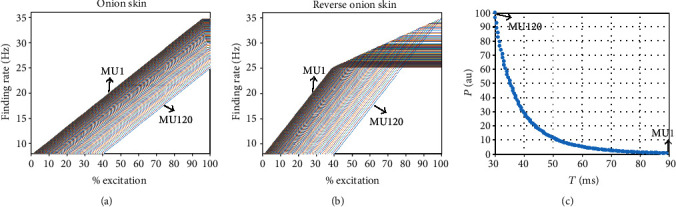
(a) The simulated relation between the excitation level and the motor unit firing rate for the “onion skin” firing strategy. (b) The simulated relation between the excitation level and the motor unit firing rate for the reverse “onion skin” firing strategy. (c) The twitch parameter assignment for the entire pool of 120 motor units.

The firing rate (FR) of each motor unit was modeled to increase linearly until the peak firing rate (PFR) was reached. To simulate the stochastic nature of motor neuron discharge, the interspike interval of the motor unit firing was modeled as a random process with a Gaussian probability distribution function. The standard deviation of the interspike interval was fixed for all motor units at 20% of the mean interspike interval as used in the original model [15]. Two motor neuron firing rate patterns were simulated. The first pattern is called the “onion skin” pattern, in which the PFR of each motor unit was inversely proportional to its RTE. Therefore, the PFRs of later recruited large motor units were lower than the early recruited small ones. On the contrary, the other pattern is called the reverse “onion skin” pattern, meaning that the PFRs of large motor units were assigned to be higher than the small motor units. In both firing strategies, the gain between the firing rate and excitatory input was set to be the same for all motor units. The FR of each motor unit at a given time was governed by Equation (2) until it reached its PFR, where *E*(*t*) was the excitatory drive.  Figure 1 shows the two different motor neuron firing patterns.

(2) $$\begin{array}{c} \text{FR}i=\text{gain}*\left[E\left(t\right)-\text{RTE}\left(\text{i}\right)\right]+\text{MFR }E\left(t\right)\geq \text{RTE}\left(i\right). \end{array}$$

### 2.2. Force Model

A motor unit twitch was modelled as a second-order critically damped impulse response (Equation (3)), where *g*_*i*,*j*_ is the force gain for discharge *j* in the *i*th motor unit, *P*_*i*_  is the peak twitch force, and *T*_*i*_ is the contraction time of the *i* th motor unit. For each motor unit, *P*_*i*_  was varied over a wide range and associated with the motor unit recruitment threshold as in Equations (4) and (5). RP is the range of peak twitch force across all the motor units, RT is the range of contraction time, ln is the natural logarithm, *i* is an index identifying each motor unit, *n* is the number of motor units, and *T*_*L*_ represents the longest contraction time. In this study, RP and RT were assigned to 100 and 3, respectively. Most motor units had lower and longer twitch forces, and a few had higher and shorter twitch forces as shown in Figure 1(c). Peak twitch force varied over a 100-fold range, and contraction time varied over 3-fold range. The highest threshold (of the last recruited motor unit) was assigned the largest force and shortest duration time.

The gain *g*_*i*,*j*_ was nonlinearly changed based on the motor unit contraction time and the interspike interval (ISI) of the discharge *j*. The gain was assigned a value of 1 when *T*_*i*_/IS*I*_*j*_ < 0.4 and then was determined as in Equation (6) when *T*_*i*_/ISI_*j*_ > 0.4. As stated in the original model [15], the greatest gain in motor unit force occurs when the ISI is equivalent to the twitch contraction time. When the motor unit firing rate exceeds a certain level, there will be no more increase in the force output. The total muscle contraction force was calculated as the linear summation of each motor unit force output.

(3) $$\begin{array}{c} f_{i,j}\left(t\right)=g_{i,j}*\frac{P_{i}∙t}{T_{i}}∙e^{1-t/T_{i}}, \end{array}$$

(4) $$\begin{array}{c} P\left(i\right)=e^{\left(\text{lnRP}/n\right)∙i}, \end{array}$$

(5) $$\begin{array}{c} T\left(i\right)=T_{L}{\left(\frac{1}{P\left(i\right)}\right)}^{1/\log_{\text{RT}}\text{RP}}, \end{array}$$

(6) $$\begin{array}{c} g_{i,j}=\frac{1-e^{-2{\left(T_{i}/{\text{ISI}}_{j}\right)}^{3}}}{T_{i}/{\text{ISI}}_{j}}\text{ when }\frac{T_{i}}{{\text{ISI}}_{j}}>0.4. \end{array}$$

### 2.3. Surface EMG Model

The muscle simulated in this study was cylindrical in shape, and the radius was 8 mm. The thickness of fat and skin layers was set to be 2.5 mm. There were totally 70,000 muscle fibers innervated by all the 120 motor neurons, which were randomly scattered in a circular territory and distributed in parallel. The density of the territory was approximately 20 fibers/mm^2^. Following an exponential function, the number of muscle fibers per motor unit was simulated to have the same range (100-fold) as the twitch force [15]. The smallest motor unit innervated 28 fibers, and the largest one innervated 2,728 fibers. The average innervation ratio was 598 fibers/motor unit. The conduction velocity was correlated to the diameter of fibers as described in [22].

**Figure 2:**
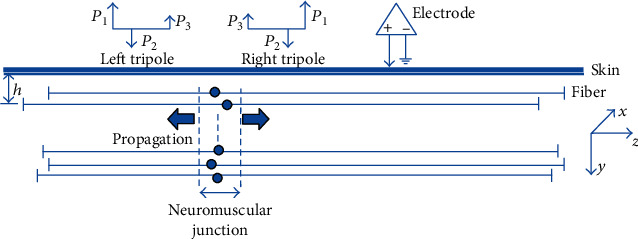
The simulated muscle fiber action potential generation and the detection system. All the muscle fibers are uniformly distributed in a cylinder at different depths *h* (*y* axis). The left or right current tripole originates from the neuromuscular junction and propagates along the direction of *z* axis, to the fiber-tendon termination.

A tripole model described in [23] was used to simulate the individual fiber action potentials in a three-dimensional muscle volume. Briefly, two action potentials generated by a fiber, modeled as two current tripoles, were originated at the innervation zone, propagated in opposite directions, and were extinct at the fiber-tendon endings (Figure 2). The monopolar signal detected by the electrode is the summation of the contribution from each of the tripoles. A motor unit action potential (MUAP) was simulated as the sum of its constituent muscle fiber action potentials.

The surface EMG signal *x*(*t*)  was then generated as a sparse combination of MUAP waveforms from all *N*  active motor units [24, 25], as described in Equation (7):

(7) $$\begin{array}{c} x\left(t\right)=\overset{N}{\underset{j=1}{\sum }}\overset{L-1}{\underset{\tau =0}{\sum }}a_{j}\left(\tau \right)s_{j}\left(t-\tau \right), \end{array}$$

where *a*_*j*_ is the MUAP waveform of the *j*th motor unit and *L* is the length of the waveform. *s*_*j*_(*t*) = ∑_*k*_*δ*(*t* − *T*_*j*_(*k*)) indicates whether the *j*th motor unit discharges at a specific time *t*, where *T*_*j*_(*k*) is the *k* th discharge time of the *j*th motor unit and *δ* represents Dirac Delta function.

### 2.4. Procedures

To investigate the effect of motor unit loss on muscle strength and the EMG-force relation, each of the parameters describing these properties was adjusted. Each time when one parameter was adjusted, the other parameters remained the same as their initial assignments.

**Figure 3:**
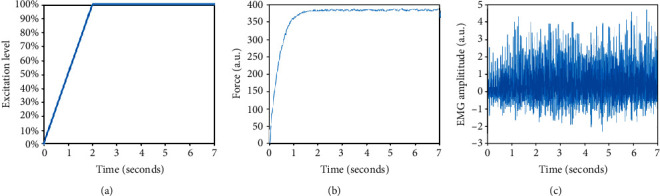
An example of (a) the excitation input simulation when the level was at 100% excitation and (b) the muscle force and (c) surface EMG outputs of the model.

The percentage of motor unit loss was set to be 20%, 40%, and 60%, respectively. Three motor unit loss patterns were simulated in this study, including the following: (1) motor unit loss restricted to the largest ones, (2) motor unit loss restricted to the smallest ones, and (3) motor unit loss without any size restriction. The force and EMG outputs at 10 excitation levels were simulated. As demonstrated in Figure 3(a), during each excitation level, the excitation drive increased linearly in the first 2 seconds and then held at a steady level for 5 seconds. The steady state excitation level ranged from 10% to 100% of maximum excitation with 10% increments. An example of simulated force and surface EMG signals is shown in Figures 3(b) and 3(c). The first 2-second transient force or EMG output was excluded from analysis, and only the steady state force and EMG signals were analyzed. The coefficient of variation (COV) of the generated force signals was calculated to estimate the force variability. The averaged rectified value (ARV) of surface EMG signals was calculated. As the force and EMG output may vary slightly because of the randomized firing time of each motor unit, 10 repetitions were simulated for each excitation level and each situation of motor unit loss. Then, the mean value of all the repetitions was calculated for each of the output parameters, including force amplitude, force COV, and ARV of surface EMG. For the EMG-force relation, the force value and surface EMG amplitude were normalized to their maximum levels. Because the variation of the simulation outcomes from different repetitions was very small, as observed in previous studies [19, 20] and confirmed in the current study, only the mean value of the 10 repetitions was reported for each simulation condition.

The model was implemented in MATLAB (MathWorks Inc., Natick, MA, USA). On average, it took approximately 10 to 15 minutes to complete simulation of one motor unit loss pattern under one firing strategy across all the excitation levels. The simulation can be run on a normal desktop or laptop computer.

## 3. Results

### 3.1. Strength of Muscle Activation

**Figure 4:**
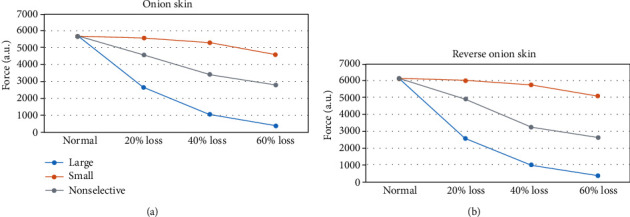
The simulated muscle force generation capacity (i.e., at 100% excitation) for different patterns and levels of motor unit loss in (a) the “onion skin” and (b) reverse “onion skin” motor unit firing strategies.

Compared with the default condition (120 motor units), motor unit loss led to reduction in muscle strength, the extent of which was related to each of the examined parameters. The muscle strength reduction caused by loss of large motor units was the greatest compared with the other two motor unit loss patterns. To examine the force capacity of the muscle, the maximum voluntary contraction force (i.e., the 100% excitation drive) was compared for different motor unit loss patterns. As shown in Figure 4, for the loss of 60% motor units restricted to the largest ones, the maximum muscle force was reduced from 5685 arbitrary units (au) to 382 au (an approximately 93.3% reduction) when the motor units followed the “onion skin” firing strategy and reduced from 6133 au to 380 au (an approximately 93.8% reduction), when the motor units followed the reverse “onion skin” firing strategy. By contrast, for the loss of 60% motor units restricted to the smallest ones, the maximum muscle force was only reduced from 5685 au to 4624 au (an approximately 18.7% reduction) when the motor units followed the “onion skin” firing strategy and reduced from 6133 au to 5090 au (an approximately 17% reduction), when the motor units followed the reverse “onion skin” firing strategy. It is worth noting that the force capacity under the motor unit reverse “onion skin” firing strategy was higher than the “onion skin” strategy.

### 3.2. Force Variability

The COV of the output force decreased substantially with increasing excitation drive under all conditions. Compared with the default condition (120 motor units), loss restricted to the largest motor units could lead to smaller COV, while motor unit loss restricted to the smallest ones or without any size restriction could lead to larger COV across all excitation levels. This was the case for both “onion skin” and reverse “onion skin” motor unit firing strategies.

**Figure 5:**
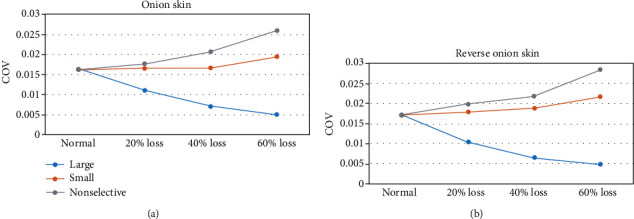
The coefficient of variation (COV) of the simulated maximum muscle force output (i.e., at 100% excitation) for different patterns and levels of motor unit loss in (a) the “onion skin” and (b) reverse “onion skin” motor unit firing strategies.

The COV of the maximum voluntary contraction force (i.e., the 100% excitation drive) was compared for different motor unit loss patterns. As shown in Figure 5(a), for the loss of 60% motor units restricted to the largest ones, the COV of the maximum muscle force was reduced by 71.4% when the motor units followed the “onion skin” firing strategy. By contrast, for the loss of 60% motor units restricted to the smallest ones or without any size restriction, the COV of the maximum muscle force was increased by 23.5% and 64.7%, respectively. The COV of the maximum muscle force followed a similar trend when motor units followed the reverse “onion skin” firing strategy, as shown in Figure 5(b).

### 3.3. EMG-Force Relation

**Figure 6:**
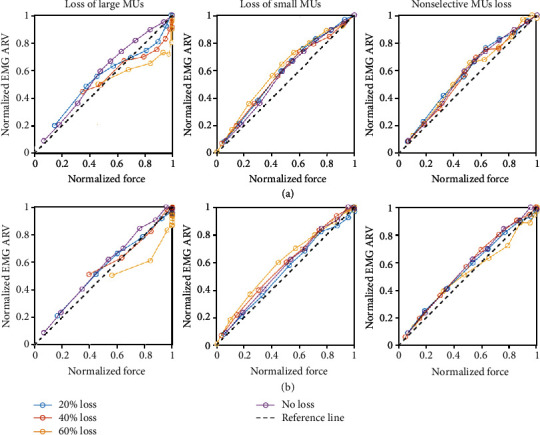
The simulated EMG-force relation for different patterns and levels of motor unit loss in (a) the “onion skin” and (b) reverse “onion skin” motor unit firing strategies.

Figure 6 shows the simulated EMG-force relation for different patterns and degrees of motor unit loss, under two motor unit firing strategies. For each constructed relation, the force and EMG were normalized to their respective maximum values at the simulated situation. For the default condition (120 motor units), the EMG-force relation could be well fit by a linear line for both motor unit firing strategies. This linear fitting of the EMG-force relation was well maintained in the situations of motor unit loss restricted to the smallest ones or without any size restriction, regardless of the degree of motor unit loss. A similar slope of the fitted line was observed for different degrees of motor unit loss restricted to the smallest ones or without any size restriction. By contrast, motor unit loss restricted to the largest ones tended to drive the approximately linear EMG-force relation to a nonlinear form, with the EMG amplitude increasing faster than force, as shown in Figure 6. This was observed for both “onion skin” and reverse “onion skin” motor unit firing strategies.

## 4. Discussion

This study implemented a model to estimate the effect of different patterns of motor unit loss on muscle force strength, variability, and the EMG-force relation. The force and EMG outputs were simulated at different levels of excitation. It was found that loss restricted to the largest motor units had the most pronounced impact on muscle strength and the EMG-force relation, while motor unit loss restricted to the smallest ones had the most pronounced impact on force variability. This was observed for both motor unit firing strategies used in the simulation.

### 4.1. Strength of Muscle Activation

As documented in previous motor unit number estimation investigations [26], progressive motor unit loss occurs after neuromuscular diseases such as amyotrophic lateral sclerosis and spinal muscular atrophy [27–29]. Transsynaptic motor neuron degeneration has also been reported in neurological injuries, causing different degrees of motor unit loss in affected muscles [1–6, 30, 31]. Motor axon degeneration following various neuropathies can also be quantified by estimating motor unit number changes [32–35]. Loss of functional motor units, especially those large ones, has a dramatic effect on muscle strength. Based on the model, twitch forces were distributed exponentially across the motor unit pool with the smallest motor unit (MU 1) assigned the smallest twitch force and the largest motor unit (MU 120) assigned the largest twitch force. Therefore, the twitch forces of one-half the motor unit population (i.e., from MU 1 to MU 60) were less than 10% of the largest motor unit's twitch force. As a result, the loss restricted to the largest motor units could cause severe muscle weakness compared with the other two motor unit loss patterns.

It was observed that for the reverse “onion skin” motor neuron firing strategy, motor unit loss restricted to the largest ones could lead more severe muscle weakness than the “onion skin” firing strategy. This is because the reverse “onion skin” firing strategy more ideally matches the motor unit contractile properties. As the excitation level increases, the discharge rate of almost all the motor units would be progressively closer to the fusion frequency, being able to reach the maximum motor unit force. However, in the “onion skin” firing strategy, the discharge rates of the largest motor units can be far below their fusion frequency even at the maximum excitation, thus compromising the maximum motor unit force generation. Consequently, the relative contribution of the largest motor units to the muscle force output is greater in the reverse “onion skin” strategy compared with the “onion skin” strategy. This results in a more severe muscle weakness if large motor units are lost in the reverse “onion” skin firing strategy.

### 4.2. Muscle Force Variability

Similar to a previous simulation study [36], the coefficient of variation (COV) of the force output decreased substantially with increasing excitation level in all simulated conditions. At submaximal excitation levels, the early recruited motor units would discharge at relatively low rates, which are below their fusion frequency. Therefore, there would be substantial ripples in the resultant motor unit force. As the excitation level increases, the motor unit firing rates start to increase, closer to the fusion frequency, gradually reducing the ripples of the individual motor unit forces. This can result in a more stable force, as suggested by the decreased COV with increasing excitation level.

Compared with the default condition, under both firing strategies, motor unit loss restricted to the largest ones could lead to a decrease in force COV, while motor unit loss restricted to the smallest ones could lead to an increase in force COV. When the lost motor units were primarily large ones, the generated force was only from small motor units even at the maximum excitation level. Given that the fusion frequency of the small motor units is low, there would be fewer ripples in the generated motor unit forces, as suggested by a decreased COV. On the other hand, when the lost motor units were primary small ones, increasing the excitation level could only activate the large motor units, whose fusion frequency is relatively high and difficult to reach. The generated force, therefore, would show more ripples as suggested by an increased COV.

### 4.3. EMG-Force Relation

The EMG-force relation has been extensively investigated in the past. A linear relation between force and the surface EMG amplitude has been reported for muscles with narrow motor unit recruitment ranges [15, 37, 38]. In the present simulation study, with the recruitment threshold set at 40% excitation, a linear EMG-force relation was also observed for both motor unit firing strategies. Interestingly, this linear relation remained in the case of motor unit loss restricted to the smallest ones or without any size restriction. If the lost motor units were primarily small ones, with increase of the excitation level, larger motor units were progressively recruited. Given relatively high fusion frequencies for the large motor units, their motor unit force continued to increase with increase of the firing rates. On the other hand, although there was an effect of EMG phase cancellation, the overall EMG amplitude also continued to increase since large MUAPs were generated with recruitment of large motor units. As a result of both force and EMG increase with excitation drive, an approximate linear EMG-force relation was maintained. There was a similar scenario for motor unit loss without size restriction since large motor units were still recruited with increase of the excitation, contributing to both force and EMG signals.

By contrast, our simulation results indicate that motor unit loss restricted to the largest ones tended to drive the EMG-force relation from a linear to nonlinear form, with EMG increasing faster than force. This is due to the fact that the remaining small motor units were assigned longer contraction time (i.e., low fusion frequency), so further increasing the motor unit firing rate with increased excitation level would not have an influence on force given that those motor units had already reached the maximum force output, and meanwhile, no larger motor units could be recruited. The increased number of MUAPs, however, increased surface EMG amplitude even there was an effect of phase cancellation. As a result, a nonlinear EMG-force relation was observed. It is worth noting that in the simulation, it was assumed that the excitation capacity still remained intact, while after a neurological injury, the excitation capacity could be impaired and consequently compromise the motor unit peak firing rates and alter the resultant EMG-force relation.

### 4.4. Limitations

As a simulation study with many assumptions used in the model, the limitations should be acknowledged. For example, the muscle fibers of each motor unit were simulated as widely scattered throughout the whole muscle, and the muscle fiber diameters of the small and large motor units were assigned the same mean value. However, motor units might locate in different regions of a muscle, with small and large motor units having different depth [39]. The size and type composition of muscle fibers belonging to small and large motor units of a muscle can also be different. Three simplified motor unit loss patterns were simulated while in real world, there is often a variable mixture of motor unit loss. A range of neurophysiological factors were not considered in the simulation, such as the excitatory and inhibitory central drive upon lower motor neurons, the interneurons input, motor unit synchronization [40], possible nonlinear effect of the individual motor unit force summation [41], and resistance to fatigue of different motor units. Of particular note, muscle fiber reinnervation as a possible compensatory process after motor unit loss was not considered in the simulation [42]. There are some other physiological changes that need to be considered in a practical situation, such as muscle atrophy, compression of motor unit recruitment range, and reduction in motor unit firing rate. All these factors may collectively alter muscle force and surface EMG signals. To address these issues, a more delicate or complicated model is required in a future study [43].

Nonetheless, by simulating three different motor unit loss patterns using the current model, we have observed their different effects on muscle weakness and changes in EMG-force relation. Motor unit loss restricted to the largest ones had the most dominant impact on the total force output and significantly changed the EMG-force relation, while loss restricted to the smallest motor units had a pronounced effect on force variability. These findings provide valuable clues for understanding experimental observations of muscle strength, force control, and EMG-force relation, especially their alterations with pathological neural or muscular changes.

## Data Availability

No experimental datasets were generated or analyzed during the current study. The simulated datasets are available from the corresponding author (PZ) on request.
